# Serologic Survey of Plague in Animals, Western Iran

**DOI:** 10.3201/eid1909.121829

**Published:** 2013-09

**Authors:** Saber Esamaeili, Kayhan Azadmanesh, Saied Reza Naddaf, Minoarisoa Rajerison, Elisabeth Carniel, Ehsan Mostafavi

**Affiliations:** Pasteur Institute of Iran, Tehran, Iran (S. Esamaeili, K. Azadmanesh, S.R. Naddaf, E. Mostsfavi);; Institut Pasteur of Madagascar, Antananarivo, Madagascar (M. Rajerison);; Institut Pasteur, Paris, France (E. Carmiel)

**Keywords:** Yersinia pestis, plague, rodents, dogs, seroepidemiology, bacteria, Iran, *Suggested citation for this article:* Esamaeili S, Azadmanesh K, Naddaf SR, Rajerison M, Carniel E, Mostafavi1 E. Serologic survey of plague in animals, western Iran [letter]. Emerg Infect Dis [Internet]. 2013 Sep [*date cited*]. http://dx.doi.org/10.3201/eid1909.121829

To the Editor: Plague has been one of the most devastating infectious diseases in human history. The etiologic agent, *Yersinia pestis*, primarily affects rodents and is usually transmitted to humans through infective flea bites. Endemic plague foci result from circulation of the plague bacillus in its rodent reservoir, the source of human plague cases (*1*). Carnivores such as dogs and foxes, which prey on rodents and eat their fresh carcasses, are valuable sentinel animals for plague serosurveillance in disease-endemic foci, although their infections are usually asymptomatic (*2*,*3*).

Plague epidemics have caused loss of human life in various parts of Iran. During 1947–1966 in western Iran, 9 human epidemics occurred and caused 156 deaths. The last case of human plague was reported in 1966 (*4*). Field investigations identified 4 *Meriones* rodent species as *Y pestis* reservoirs; 2 were resistant (*M. persicus* and *M. libycus*), and the other 2 (*M. tristrami* and *M. vinogradovi*) were susceptible to death from infection (*4*,*5*). The epidemiologic investigations demonstrated a 3–4 year plague epizootic cycle in Iran (*5*). The last official report of plague in rodents in Iran dates back to 1978, in Sarab County in the East Azarbaijan Province (*6*). Plague surveillance was ignored for more than 3 decades and then restarted in 2011 in Iran.

This study was designed to investigate plague among resident animals in western Iran, specificallyregion localities along the border between the Kurdistan and Hamadan Provinces, where plague in wildlife has been repeatedly reported (enclosed by 47.900° and 48.284° north latitude and 35.4616° and 35.7829° east longitude). The epidemiologic team was based at the Akanlu Research Center of the Pasteur Institute of Iran, in a village ≈100 km from Kabudar Ahang, Hamadan Province, at an altitude of ≈1,600 m. The study was conducted during June–September in 2011 and 2012. In 2011, a large area (2,000 km^2^) was selected and, because only 1 *Y. pestis*–positive dog sample was found, in 2012, , the study area was reduced to 1,200 km^2^ and confined to localities in which the *Y. pestis*–positive dog sample was identified the previous year; 3 additional *Y. pestis*–positive dogs and 1 *Y. pestis*–positive rodent were found in 2012.

The average number of traps used per night per locality was 13. A total of 46 rodents were entrapped from 26 localities in 998 traps (4.61% success) during the first year, and 52 rodents were captured in 30 localities in 1,164 traps (4.46% success) during the second year. They were mostly members of the *Meriones* genus, although a few *Microtus socialis irani* and 1 *Ellobius lutescens* rodents were also caught (Table). A total of 281 fleas were collected on 70.41% of trapped rodents (Table), corresponding to an average flea index of 4.10 for infested rodents. All fleas were *Xenopsylla* spp. ELISA was performed as described (*7*) to detect antibodies against *Y. pestis* F1 capsular antigen. Samples positive by ELISA were confirmed by using the inhibition ELISA method (*8*). Of 98 trapped rodents, 1 (1.02%) had IgG against F1 (Table), an *M. persicus* jird caught in 2012.

**Table:** Number of fleas and serum samples with *Yersinia pestis* F1 antibody collected from various animals during field investigations, Iran, 2011–2012*

Animal/species	No. serum samples	No. serum samples with antibodies against F1	No. fleas collected
Rodents			
2011			
* Meriones persicus*	39	0	33
* Meriones vinogradovi*	3	0	0
* Microtus socialis irani*	4	0	1
* 2012*			
* Meriones persicus*	24	1	146
* Meriones libycus*	26	0	101
* Microtus socialis irani*	1	0	0
* Ellobius lutescens*	1	0	0
Total	98	1	281
Sheepdogs			
2011			
ND	58	1	ND
2012			
ND	59	3	ND
Total	117	4	
Miscellaneous			
Jackals			
2011			
* Canis aureus*	2	0	ND
2012			
* C. aureus*	0	0	ND
Foxes			
2011			
* Vulpes vulpes*	1	0	ND
2012			
* V. vulpes*	1	0	ND
Rabbits			
2011			
* Lepus capensis*	1	0	ND
2012			
* L. capensis*	7	0	ND
Hedgehogs			
2011	0	0	
ND			ND
2012	1	1	
ND			ND
Total	14	0	

*ND, not determined.

Sheepdogs that lived in the study areas were also used as sentinel animals. Blood samples were collected from 58 sheepdogs in 15 villages in 2011 and from 59 sheepdogs in 8 villages in 2012. Of 117 dog serum samples analyzed, 4 (3.42%) had IgG titers against F1, 1 in 2011 and the other 3 in 2012 (Table). Finally, wild animals such as jackals, foxes, rabbits, and hedgehogs were hunted in the study areas, and blood samples were taken immediately. None of the serum samples obtained from 3 foxes, 2 jackals, 8 rabbits, and 1 hedgehog had IgG against F1 (Table).

Because a well-established plague focus existed in Iranian Kurdistan, with animal cases occurring until 1978 (*9*), complete extinction of this focus is most unlikely. Our study demonstrates that animal reservoirs (*Meriones* rodents) and flea vectors (*Xenopsylla* spp*.*) shown to be central to the plague ecologic cycle in Iran still are found in high numbers in a previously active focus. The fact that 70% of trapped rodents were infested with fleas, with an average *Xenopsylla* spp. index of 4.10, may be considered as circumstances most favorable for the onset of plague epizootics. Furthermore, the detection of *Y. pestis*–specific IgG in 1.02% of trapped rodents and 3.42% of sentinel dogs is highly suggestive of active circulation of *Y. pestis* in its natural animal reservoir. Because *Y. pestis* antibodies last only for ≈6 months in dogs (*2*), seropositivity of these dogs indicates newly acquired infections.

This fact that *Y. pestis*–-positive animals were found over the 2-year surveillance period suggests that this area could be an active plague focus. Therefore, although no official reports of human plague in Iran have been made since 1966, this study indicates that the epidemiologic conditions needed to trigger an outbreak have been met. It is thus of utmost importance to maintain and strengthen the health system with plague surveillance in western Iran.
